# Vestibular Involvement in Systemic Autoimmune and Rheumatologic Diseases: A Systematic Review and GRADE-Based Assessment

**DOI:** 10.3390/jcm15082841

**Published:** 2026-04-09

**Authors:** Juan C. Amor-Dorado, Miguel A. González-Gay

**Affiliations:** 1 Otorhinolaryngology Division, Hospital Can Misses, 07800 Ibiza, Spain; 2 Rheumatology Division, IIS–Fundación Jiménez Díaz, 28015 Madrid, Spain; miguelaggay@hotmail.com; 3 Department of Medicine, School of Medicine, University of Cantabria, 39011 Santander, Spain

**Keywords:** vestibular dysfunction, autoimmune diseases, rheumatologic diseases, systemic sclerosis, giant cell arteritis, benign paroxysmal positional vertigo, balance disorders, vestibular assessment, systematic review, GRADE

## Abstract

**Background:** Vestibular symptoms and objective vestibular dysfunction have been reported in patients with autoimmune and rheumatologic diseases, but available evidence remains fragmented and methodologically heterogeneous. Previous studies have often addressed audiovestibular involvement as a combined entity, limiting disease-specific interpretation of vestibular outcomes. **Methods:** A PRISMA 2020-based systematic review was conducted using predefined eligibility criteria targeting vestibular outcomes in autoimmune and systemic rheumatologic diseases. Observational studies reporting vestibular symptoms and/or objective vestibular test results were included. Vestibular data were extracted even when studies reported combined audiovestibular outcomes. Certainty of evidence was assessed using the GRADE approach. **Results:** Twenty-seven studies were included in the qualitative synthesis, comprising 14 primary observational studies and 13 reviews. Vestibular involvement was reported across multiple diseases, including systemic sclerosis, giant cell arteritis, ankylosing spondylitis, psoriatic arthritis, Behçet disease, primary Sjögren syndrome, rheumatoid arthritis, systemic lupus erythematosus, antiphospholipid syndrome, and vasculitic disorders. Objective vestibular abnormalities were most frequently identified using caloric testing, balance integration measures, videonystagmography, and video head impulse testing. Systemic sclerosis and giant cell arteritis showed more consistently reported vestibular findings, although heterogeneity in assessment methods precluded quantitative synthesis. **Conclusions:** Vestibular involvement occurs across autoimmune and systemic inflammatory diseases, but overall certainty of evidence remains limited. Standardized vestibular assessment and longitudinal studies are needed to better define disease-specific vestibular phenotypes.

## 1. Introduction

Systemic autoimmune and rheumatologic diseases are characterized by immune-mediated mechanisms that may affect multiple organs, including the inner ear. While cochlear involvement has been extensively investigated, vestibular manifestations remain comparatively underrecognized and inconsistently characterized. Symptoms such as vertigo, dizziness, and imbalance have been reported in several autoimmune conditions, yet their true prevalence, patterns of vestibular dysfunction, and clinical implications remain insufficiently defined [1]. Vestibular manifestations in these disorders may include episodic or chronic vertigo, positional vertigo, imbalance, and nonspecific dizziness, which can reflect both peripheral vestibular dysfunction and central vestibular involvement. In some patients, abnormalities are detected only through objective vestibular testing, such as caloric testing, videonystagmography, video head impulse testing, or vestibular evoked myogenic potentials, suggesting that vestibular dysfunction may remain subclinical in a proportion of cases [1].

Previous literature has frequently examined inner ear involvement within a combined audiovestibular framework, often emphasizing hearing loss or autoimmune inner ear disease. As a result, vestibular findings have commonly been reported as secondary outcomes or without disease-specific stratification, thereby limiting the ability to identify consistent patterns of vestibular dysfunction across systemic autoimmune disorders [1,2].

From a pathophysiological perspective, the vestibular system may be particularly vulnerable to systemic immune-mediated processes. The labyrinthine structures depend on a highly specialized microvascular supply, and vestibular sensory epithelia are sensitive to inflammatory and ischemic injury. Immune-mediated mechanisms affecting the labyrinthine circulation, endolymphatic homeostasis, or vestibular nerve pathways may therefore contribute to both peripheral and central patterns of vestibular dysfunction. Nevertheless, vestibular manifestations have historically received less attention than cochlear involvement in autoimmune diseases, resulting in important gaps in clinical characterization and epidemiological understanding [3,4,5,6,7,8,9]. Among systemic autoimmune diseases, systemic sclerosis has shown relatively consistent evidence of vestibular involvement in case–control studies demonstrating objective abnormalities in caloric testing and postural integration [3,4,5]. Similar associations have been described in giant cell arteritis, ankylosing spondylitis, psoriatic arthritis, rheumatoid arthritis, primary Sjögren syndrome, and selected vasculitides; however, substantial heterogeneity in study design and vestibular assessment methods persists across studies [6,7,8,9,10,11,12,13,14,15,16,17,18].

Increasing evidence suggests that immune-mediated mechanisms—including microvascular compromise, inflammatory injury, and immune complex deposition—may contribute to vestibular dysfunction in systemic autoimmune diseases. However, the available data remain fragmented, and methodological variability across studies limits the interpretation and generalizability of reported findings [4,19,20,21,22].

The aim of the present study was to systematically review and qualitatively synthesize primary evidence on vestibular involvement in systemic autoimmune and rheumatologic diseases, and to appraise the certainty of evidence across disease categories using the GRADE framework [23].

## 2. Materials and Methods

This systematic review was conducted and reported in accordance with the PRISMA 2020 statement [24] (Supplementary Materials).

### 2.1. Search Strategy

A comprehensive literature search was performed in PubMed/MEDLINE, Embase, Web of Science, and the Cochrane Library from database inception until the final search date (December 2025). The search strategy combined controlled vocabulary and free-text terms related to vestibular involvement and autoimmune diseases, including: vestibular, vertigo, dizziness, balance, autoimmune, rheumatologic, systemic sclerosis, vasculitis, giant cell arteritis, ankylosing spondylitis, psoriatic arthritis, Behçet, Sjögren, rheumatoid arthritis, and systemic lupus erythematosus. Reference lists of relevant reviews and included articles were manually screened to identify additional eligible studies. No artificial intelligence tools were used for data analysis, interpretation, or generation of scientific content.

### 2.2. Eligibility Criteria

Studies were included if they met the following criteria:

(1) observational design (cohort, case–control, or cross-sectional);
(2) evaluation of patients with autoimmune, autoinflammatory, or systemic rheumatologic diseases;
(3) reporting vestibular symptoms or objective vestibular test results;
(4) availability of extractable vestibular data.

Studies were excluded if they:

(1) focused exclusively on auditory outcomes;
(2) were case reports, conference abstracts, editorials, or animal studies;
(3) lacked sufficient methodological detail;
(4) reported overlapping patient cohorts without novel vestibular data.

When studies reported combined audiovestibular outcomes, only vestibular-related data were extracted and analyzed, in line with previous methodological recommendations [1,2].

### 2.3. Study Selection

**Figure 1:**
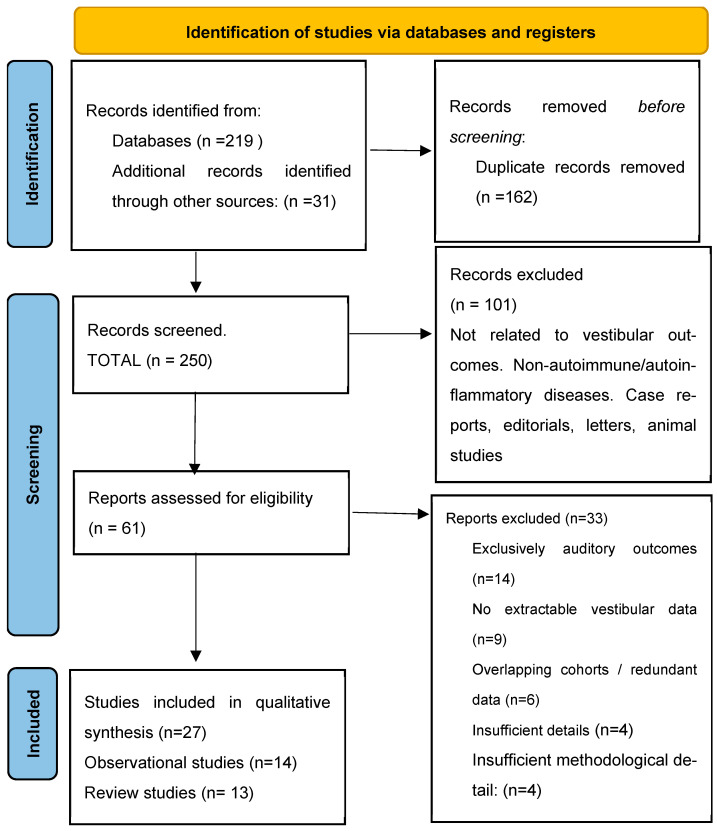
PRISMA 2020 flow diagram illustrating study selection for the systematic review of vestibular involvement in autoimmune and systemic rheumatologic diseases.

After duplicate removal, titles and abstracts were independently screened for eligibility. Full-text articles were retrieved and assessed when abstracts suggested potential relevance. Two reviewers independently performed the screening, and disagreements were resolved through discussion and consensus. The study selection process is summarized in the PRISMA flow diagram (Figure 1) [24].

### 2.4. Data Extraction

For each included primary study, the following data were extracted: first author, year of publication, disease category, study design, sample size, vestibular assessment methods, vestibular outcomes, and main findings. Reviews were analyzed separately to contextualize the primary evidence. Data extraction was performed using a standardized form and cross-checked for accuracy.

### 2.5. Risk-of-Bias Assessment

The risk of bias was qualitatively assessed for primary studies based on the study design, sample size, selection criteria, and completeness of vestibular outcome reporting, following methodological principles commonly applied to observational clinical research.

### 2.6. Certainty of Evidence (GRADE)

The certainty of evidence for vestibular involvement was evaluated using the Grading of Recommendations Assessment, Development and Evaluation (GRADE) framework. The assessment was conducted at the level of disease-specific bodies of evidence rather than individual studies, in accordance with GRADE guidance for systematic reviews.

Because all included studies were observational in design (cohort or case–control), the certainty of the evidence for each disease category was initially rated as low. The certainty rating was then evaluated across the following GRADE domains: risk of bias, inconsistency, indirectness, imprecision, and other considerations (including magnitude of effect and coherence of findings).

Two reviewers independently assessed the certainty of evidence. Each reviewer performed a structured evaluation of the predefined GRADE domains for every disease category. Discrepancies were discussed in detail and resolved by consensus. All disagreements were resolved through discussion, and no external adjudication was required.

Risk-of-bias considerations were informed by the methodological characteristics of the included studies, including adequacy of control groups, clarity of inclusion criteria, use of standardized or objective vestibular testing, and completeness of outcome reporting. Inconsistency was evaluated by examining variability in vestibular outcomes and heterogeneity of diagnostic methods across studies within each disease category. Indirectness was considered when vestibular outcomes were secondary endpoints or when assessment methods lacked standardization. Imprecision was qualitatively appraised, taking into account sample size, variability of findings, and strength of reported associations.

Upgrading the certainty of evidence was considered only when consistent and methodologically robust findings were observed across multiple independent studies using objective vestibular measures. Final certainty judgments were assigned per disease category and are presented in Section 3.

## 3. Results

### 3.1. Study Selection

The systematic literature search identified a total of 27 eligible studies for qualitative synthesis after removal of duplicates and application of predefined inclusion and exclusion criteria (Figure 1). These comprised 14 primary observational studies addressing vestibular involvement in specific diseases and 13 reviews providing clinical, epidemiological, or mechanistic context. No randomized controlled trials were identified. All included studies were published in peer-reviewed journals.

### 3.2. Characteristics of Included Studies

The characteristics of the 14 primary observational studies are summarized in Table 1. Most studies used a case–control or cross-sectional design and included adult patients with established autoimmune or systemic rheumatologic diseases. Sample sizes varied widely across studies, ranging from small cohorts to larger disease-specific populations.

The 13 review articles included narrative reviews, systematic reviews, and one systematic review with meta-analysis. These reviews addressed vestibular involvement either directly or as part of broader audiovestibular or immune-mediated inner ear frameworks and are summarized in Table 2.

### 3.3. Vestibular Involvement Across Autoimmune and Rheumatologic Diseases

Vestibular symptoms and objective vestibular abnormalities were reported across a wide spectrum of diseases, including systemic sclerosis, giant cell arteritis, ankylosing spondylitis, psoriatic arthritis, Behçet disease, primary Sjögren syndrome, rheumatoid arthritis, systemic lupus erythematosus, antiphospholipid syndrome, ANCA-associated vasculitis, sarcoidosis, and Cogan syndrome.

Across studies, vestibular involvement was evaluated using a combination of clinical symptom assessment (vertigo, dizziness, imbalance, and benign paroxysmal positional vertigo) and objective vestibular testing, most commonly caloric testing, videonystagmography (VNG), clinical tests of sensory integration and balance (CTSIB), and video head impulse testing (vHIT). The choice of vestibular assessment methods varied substantially between studies, limiting direct comparisons.

In systemic sclerosis, multiple independent studies reported a higher prevalence of vertigo, balance impairment, and benign paroxysmal positional vertigo compared with control populations. Objective abnormalities were frequently identified using caloric testing and balance integration measures. These findings were consistently reported across different cohorts.

In giant cell arteritis, vestibular involvement was characterized predominantly by an increased prevalence of benign paroxysmal positional vertigo (BPPV), confirmed by positional testing. Although objective vestibular testing beyond positional maneuvers was limited, findings were reproducible across independent observational studies.

In ankylosing spondylitis and psoriatic arthritis, vestibular involvement was mainly reflected by impaired balance performance and increased rates of positional vertigo. Studies frequently relied on clinical balance tests and posturography, with fewer studies incorporating semicircular canal-specific vestibular assessments.

In Behçet disease, objective vestibular abnormalities were identified using vHIT and oculomotor or positional maneuvers, suggesting peripheral semicircular canal involvement in selected cohorts. In primary Sjögren syndrome, vestibular abnormalities were often subclinical and detected through physiological vestibular testing rather than overt vestibular symptoms.

In rheumatoid arthritis, vestibular findings were heterogeneous, with studies reporting both peripheral vestibular hypofunction and central-type oculomotor abnormalities. Similar heterogeneity was observed in systemic lupus erythematosus, where vestibular involvement was primarily symptom-based and objective testing was inconsistently applied.

Vestibular involvement in antiphospholipid syndrome, ANCA-associated vasculitis, sarcoidosis, and Cogan syndrome was reported in a limited number of studies, typically small observational cohorts or case series, precluding robust disease-specific comparisons.

### 3.4. Certainty of Evidence (GRADE Assessment)

The certainty of evidence for vestibular involvement was assessed using the GRADE framework, considering the overall body of evidence available for each disease (Table 3). For systemic sclerosis and giant cell arteritis, certainty of evidence was rated as moderate at best, within the limitations inherent to observational study designs and heterogeneous vestibular assessment methods.

For ankylosing spondylitis, psoriatic arthritis, Behçet disease, primary Sjögren syndrome, rheumatoid arthritis, systemic lupus erythematosus, and antiphospholipid syndrome, certainty of evidence was rated as low, primarily due to small sample sizes, methodological heterogeneity, and limited use of standardized vestibular testing. For ANCA-associated vasculitis, sarcoidosis, and Cogan syndrome, the certainty of the evidence was rated as very low, reflecting sparse and predominantly descriptive data.

## 4. Discussion

This systematic review indicates that vestibular involvement has been reported across a broad range of systemic autoimmune and rheumatologic diseases, although the available evidence remains heterogeneous and largely observational. By specifically focusing on vestibular outcomes, the present study addresses an important limitation of previous audiovestibular syntheses, in which vestibular findings were frequently embedded within combined audiovestibular outcomes or reported using heterogeneous diagnostic approaches. The results of this review indicate that vestibular symptoms and objective vestibular abnormalities have been described in several autoimmune conditions; however, differences in study design, sample size, and vestibular assessment methods limit direct comparisons and prevent quantitative synthesis. These findings highlight the need for more standardized vestibular evaluation protocols and longitudinal studies to better define disease-specific vestibular phenotypes in systemic autoimmune disorders [1,2,6,8].

Across the included conditions, systemic sclerosis (SSc) and giant cell arteritis (GCA) were the diseases in which vestibular findings were most consistently reported across multiple independent primary studies. However, they lacked high-level causal evidence. In SSc, case–control studies reported higher rates of vertigo and balance impairment, as well as an increased prevalence of benign paroxysmal positional vertigo (BPPV), compared with controls [3,5,9]. A recent systematic review and meta-analysis supports a higher frequency of vertigo in SSc. However, objective vestibular outcomes could not be quantitatively pooled because of heterogeneous testing protocols and outcome definitions [3]. Collectively, these data suggest a reproducible association between SSc and vestibular symptoms, while emphasizing the need for standardized vestibular endpoints to enable robust comparisons and pooling.

In GCA, observational studies reported a higher prevalence of BPPV confirmed by positional testing [9,25]. Although these findings do not establish causality, they align with narrative syntheses of systemic vasculitis, which propose that ischemic involvement of the labyrinthine circulation may contribute to vestibular syndromes in this setting [25]. Importantly, the certainty of evidence should be interpreted within the limitations of observational designs, and mechanistic explanations should be framed as plausible rather than definitive [25].

Evidence from spondyloarthritis-related diseases suggests a tendency toward vestibular/balance involvement. In ankylosing spondylitis and psoriatic arthritis, studies using sensory integration and balance measures, and, in some cases, posturography, reported higher rates of balance impairment and positional vertigo compared with controls [10,11,26,27]. While these findings are clinically relevant, differences in vestibular assessment strategies and the frequent reliance on nonspecific balance endpoints limit disease-specific phenotyping and cross-study comparability. More recent studies have expanded objective vestibular assessment to additional autoimmune diseases. In Behçet disease, abnormalities detected using video head-impulse testing and oculomotor/positional maneuvers suggest peripheral semicircular canal involvement in selected cohorts [12]. In primary Sjögren syndrome, vestibular abnormalities were often subclinical and identified through physiological testing [13]. In rheumatoid arthritis, vestibular findings were heterogeneous across studies, with both peripheral hypofunction and central-type oculomotor abnormalities reported, likely reflecting differences in patient selection and testing protocols [14]. Vestibular involvement has also been described in systemic lupus erythematosus, antiphospholipid syndrome, ANCA-associated vasculitis, sarcoidosis, and Cogan syndrome, although the available evidence is limited and heterogeneous [15,16,17,18].

Ménière’s disease was not considered a systemic autoimmune disorder in this review. However, it was included as a related clinical model of recurrent vertigo with documented autoimmune comorbidity. Large observational data indicate an increased prevalence of systemic autoimmune diseases among patients with Ménière’s disease, supporting an autoimmune background in a subset and highlighting potential immune contributions to vertigo persistence [28]. These findings provide contextual support for immune-mediated vestibular symptom chronicity, while remaining distinct from disease-specific vestibular testing evidence.

The clinical concept of immune-mediated inner ear involvement predates modern vestibular testing and was historically framed as autoimmune inner ear disease (AIED). Classical reviews described frequent vestibular symptoms and a relevant coexistence with systemic autoimmune disorders [3]. More recently, mechanistic reviews have emphasized the role of resident macrophages and innate immune signaling within the inner ear, providing biological plausibility for immune-mediated effects on vestibular end organs, although these data do not confirm clinical vestibular dysfunction [20]. In parallel, biomarker-focused studies highlight the ongoing lack of sensitive and specific diagnostic immune biomarkers for routine clinical use, which may partly explain the heterogeneity of clinical phenotypes and outcomes reported across studies [21]. Recent narrative syntheses on AIED pathogenesis and treatment similarly underscore that therapeutic responses to corticosteroids or immunosuppressive agents are variable and largely supported by observational evidence, reinforcing the need for standardized vestibular phenotyping and longitudinal designs [6,8,29].

Overall, applying the GRADE framework indicates that the certainty of the evidence is moderate at best for a limited subset of conditions and low to very low for most diseases, primarily due to observational study designs, limited sample sizes, and inconsistency in vestibular assessment methodologies [23]. Future studies should prioritize standardized vestibular testing protocols (including clearly defined symptom definitions and objective endpoints), longitudinal follow-up, and transparent reporting of disease activity and treatments to clarify disease-specific vestibular phenotypes and their clinical implications.

## 5. Limitations of the Study

Several limitations of this review should be acknowledged.

First, the available evidence is predominantly derived from observational studies, mainly case–control and cohort designs. The absence of randomized controlled trials and robust longitudinal investigations limits the ability to establish causal relationships between systemic autoimmune diseases and vestibular dysfunction.

Second, many included studies involved relatively small sample sizes, particularly in rare autoimmune conditions, which reduced statistical precision and may have increased uncertainty in the reported associations.

Third, substantial methodological heterogeneity was observed across studies. Variability in study design, diagnostic criteria, vestibular assessment protocols, and outcome definitions limited comparability and precluded quantitative synthesis. Vestibular evaluation ranged from symptom-based clinical assessment to objective instrumental testing (e.g., caloric testing, video head impulse testing, vestibular evoked myogenic potentials, and posturography), without consistent standardization.

Additionally, vestibular manifestations were frequently reported as secondary outcomes rather than primary study endpoints, potentially leading to underestimation or non-systematic assessment of vestibular involvement.

Publication bias cannot be excluded, as studies reporting significant associations may be more likely to be published.

Taken together, these limitations contribute to an overall low-to-moderate certainty of evidence across most disease categories, as reflected in the GRADE assessment.

Although vestibular manifestations in systemic autoimmune diseases have been previously reported, this review provides a focused and structured synthesis dedicated specifically to vestibular involvement. By distinguishing subjective symptoms from objectively confirmed dysfunction and systematically documenting the vestibular tests employed, we clarify disease-specific patterns and highlight methodological heterogeneity across studies. The formal application of the GRADE framework further strengthens this work by contextualizing findings according to their certainty of evidence.

Importantly, this review identifies key methodological gaps—small sample sizes, inconsistent assessment protocols, and limited longitudinal data—that constrain current knowledge and explain the predominantly low-to-moderate certainty of the evidence.

## 6. Conclusions

Vestibular involvement has been reported across a wide spectrum of systemic autoimmune and rheumatologic diseases. However, the overall certainty of evidence remains limited and is predominantly based on observational data. Among the conditions reviewed, systemic sclerosis and giant cell arteritis show the most consistently reported vestibular findings across independent studies, although the certainty of evidence should be interpreted as moderate at best and within the limitations inherent to observational designs. For most other diseases, evidence remains low or very low due to heterogeneous methodologies, small sample sizes, and inconsistent vestibular assessment strategies. Future research should prioritize standardized vestibular testing protocols, longitudinal designs, and transparent reporting of disease activity and treatment exposure to better define disease-specific vestibular phenotypes and their clinical implications.

## Figures and Tables

**Table 1:** Primary studies reporting vestibular involvement in autoimmune and rheumatologic diseases.

Author (Year)	Disease	Study Design	Participants (Patients/Controls)	Vestibular Tests	Vestibular Outcomes	Main Findings	Risk of Bias
Amor-Dorado et al. (2003) [25]	Giant cell arteritis	Prospective cohort	44/44	Positional tests; caloric test	Vertigo; peripheral vestibulopathy	Vestibular dysfunction more frequent than controls	Moderate
Amor-Dorado et al. (2004) [9]	Giant cell arteritis	Case–control	44/44	Dix–Hallpike maneuver	BPPV	Strong association between GCA and BPPV	Moderate
Amor-Dorado et al. (2008a) [4]	Limited systemic sclerosis	Case–control	35/59	Caloric test; CTSIB; oculography	Chronic imbalance	Objective vestibular abnormalities more frequent	Moderate
Amor-Dorado et al. (2008b) [5]	Systemic sclerosis	Case–control	42/74	Dix–Hallpike; CTSIB	BPPV; postural instability	Increased BPPV prevalence and abnormal sensory integration	Moderate
Amor-Dorado et al. (2011a) [10]	Ankylosing spondylitis	Case–control	59/46	Caloric test; CTSIB	Dizziness; imbalance	Peripheral hypofunction and impaired balance integration	Moderate
Amor-Dorado et al. (2011b) [26]	Ankylosing spondylitis	Case–control	59/46	Dix–Hallpike; CTSIB	BPPV	Higher BPPV prevalence than controls	Moderate
Amor-Dorado et al. (2014) [11]	Psoriatic arthritis	Case–control	60/60	Caloric test; CTSIB	Dizziness; imbalance	Significant vestibular dysfunction vs. controls	Moderate
Amor-Dorado et al. (2017) [27]	Psoriatic arthritis	Case–control	60/60	Oculography; CTSIB; CDP	Postural instability	Objective balance abnormalities	Moderate
Gázquez et al. (2011) [28]	Ménière’s disease with autoimmune comorbidity	Multicenter cohort	690/–	AAO–HNS criteria	Episodic vertigo	Autoimmune comorbidity linked to persistent vertigo	Moderate
Karataş et al. (2007) [15]	Systemic lupus erythematosus	Observational	28/–	Clinical assessment	Vertigo; dizziness	High frequency of vestibular symptoms	High
Ertugrul et al. (2019) [12]	Behçet disease	Prospective case–control	31/31	vHIT; head-shake test; DHI	Peripheral dysfunction	Horizontal canal dysfunction and nystagmus more frequent	Moderate
Özkırış et al. (2014) [14]	Rheumatoid arthritis	Prospective case–control	81/81	VNG; caloric; positional tests	Central and peripheral dysfunction	VNG abnormalities (38.3%); canal paresis (13.6%)	Moderate
Ulusoy et al. (2022) [13]	Primary Sjögren syndrome	Case–control	35/35	vHIT; cVEMP; oVEMP	Subclinical dysfunction	Reduced vHIT gain; abnormal VEMP latencies	Moderate
Morita et al. (2017) [17]	ANCA-associated vasculitis	Observational study	31/–	Audiovestibular assessment	Vestibular dysfunction	Inner ear involvement in ANCA vasculitis	Moderate

Risk-of-Bias Summary: Overall, most studies presented a moderate risk of bias, primarily due to observational design, small sample size, and limited control of confounding variables. A high risk of bias was identified in studies relying exclusively on non-standardized clinical assessment without objective vestibular testing (Karataş et al. [15]). Abbreviations: AAO–HNS: American Academy of Otolaryngology–Head and Neck Surgery. BPPV: benign paroxysmal positional vertigo. CDP: computerized dynamic posturography. CTSIB: Clinical Test of Sensory Interaction and Balance. DHI: Dizziness Handicap Inventory. vHIT: video head impulse test. VNG: videonystagmography. VEMP: vestibular evoked myogenic potentials.

**Table 2:** Reviews addressing vestibular involvement in autoimmune and systemic rheumatologic diseases.

Ref	Author (Year)	Type of Review	Diseases Included	Vestibular Scope	Main Contribution
[1]	Ralli et al. (2018)	Narrative clinical review	SLE, RA, Sjögren, Behçet, vasculitis, Cogan syndrome, sarcoidosis	Clinical vestibular symptoms	Highlights the frequency and underrecognition of vestibular symptoms
[2]	Girasoli et al. (2018)	Narrative clinical review	Autoimmune disorders	Clinical vestibular syndromes	Overview of immune-mediated vertigo
[6]	Bovo et al. (2006)	Narrative clinical review	Autoimmune inner ear disease	Vertigo, imbalance, episodic vertigo	Classical clinical description of AIED
[29]	Amor-Dorado et al. (2009)	Narrative clinical review	Systemic vasculitides and connective tissue diseases	Vertigo, nystagmus, BPPV	Early synthesis linking vasculitis and vestibular involvement
[8]	Breslin et al. (2020)	Systematic review	Autoimmune inner ear disease	Secondary vestibular outcomes	Vestibular data inconsistently reported
[28]	Gázquez et al. (2011)	Narrative review/cohort synthesis	Ménière’s disease + autoimmune comorbidity	Recurrent vertigo	Increased autoimmune prevalence in Ménière’s disease
[7]	Athanasopoulos et al. (2024)	Narrative mechanistic review	Autoimmune/autoinflammatory diseases	Mechanistic	Immune-inflammatory and vascular mechanisms
[20]	Miwa and Okano (2022)	Narrative mechanistic review	Autoimmune inner ear disease	Translational	Macrophage-mediated immune mechanisms
[19]	Li et al. (2023)	Narrative mechanistic review	Autoimmune inner ear disorders	Mechanistic	Immune microenvironment pathways
[3]	Salvador et al. (2025)	Systematic review and meta-analysis	Systemic sclerosis	Clinical vestibular outcomes	Increased vertigo prevalence; high heterogeneity
[16]	Chen et al. (2024)	Systematic review	Antiphospholipid syndrome	Clinical vestibular outcomes	Evidence linking APS and recurrent vertigo
[18]	Mazlumzadeh et al. (2007)	Narrative review	Cogan syndrome	Clinical audiovestibular involvement	immune-mediated vestibular and ocular manifestations
[30]	Colvin, IB (2006)	Narrative review	Sarcoidosis	Clinical audiovestibular manifestations	Describes vestibular involvement (vertigo, imbalance) in sarcoidosis

AIED, autoimmune inner ear disease; APS, antiphospholipid syndrome; BPPV, benign paroxysmal positional vertigo; GCA, giant cell arteritis; PAN, polyarteritis nodosa; RA, rheumatoid arthritis; SLE, systemic lupus erythematosus; SSc, systemic sclerosis.

**Table 3:** Certainty of evidence for vestibular involvement assessed using the Grading of Recommendations Assessment, Development and Evaluation (GRADE) approach. Certainty ratings were assigned by disease, based on the overall body of relevant evidence available for each autoimmune or rheumatologic condition.

Disease	Vestibular Outcomes	Evidence Base	Main Limitations	Certainty of Evidence (GRADE)
Systemic sclerosis	Vertigo, balance impairment, BPPV	Multiple observational case–control studies; systematic review and meta-analysis	Observational designs; heterogeneous vestibular testing; lack of longitudinal data	Moderate at best, within observational limitations
Giant cell arteritis	BPPV, vertigo	Observational cohort and case–control studies	Small sample sizes; limited objective vestibular testing	Moderate at best, within observational limitations
Ankylosing spondylitis	Balance impairment, BPPV	Observational case–control studies	Nonspecific balance endpoints; heterogeneous methods	Low
Psoriatic arthritis	Balance impairment	Observational case–control studies	Small cohorts; limited vestibular specificity	Low
Behçet disease	Vestibular hypofunction, abnormal vHIT	Observational studies	Limited number of studies; inconsistent outcomes	Low
Primary Sjögren syndrome	Subclinical vestibular abnormalities	Single observational study	Small sample size; lack of replication	Low
Rheumatoid arthritis	Peripheral and central vestibular abnormalities	Observational studies	Conflicting findings; heterogeneous testing	Low
Systemic lupus erythematosus	Vertigo, vestibular dysfunction	Observational studies	Symptom-based outcomes; limited objective data	Low
Antiphospholipid syndrome	Recurrent vertigo	Observational studies; systematic review	Sparse primary data; indirect vestibular outcomes	Low
ANCA-associated vasculitis	Vestibular dysfunction	Small observational series	Very limited evidence	Very low
Sarcoidosis	Vestibular involvement	Case series	Rare condition; non-comparative data	Very low
Cogan syndrome	Vestibular dysfunction	Observational studies	Rare disease; mixed audiovestibular outcomes	Very low

BPPV, benign paroxysmal positional vertigo; GRADE, Grading of Recommendations Assessment, Development and Evaluation; vHIT, video head impulse test.

## Data Availability

This study is based on previously published data. No new data were generated or analyzed.

## Supplementary Materials

The following supporting information can be downloaded at: https://www.mdpi.com/article/10.3390/jcm15082841/s1, PRISMA 2020 Checklist.
